# Evolutionary dynamics of the calcium/cation antiporter superfamily in Brassicaceae: codon usage, selection pressure, and *BnCaCAs* role in abiotic stress response

**DOI:** 10.3389/fpls.2025.1506461

**Published:** 2025-07-08

**Authors:** Amin Abedi, Mohadece Pourkarimi Daryakenari, Fatemeh Zare, Somayeh Allahi, Zahra Hajiahmadi

**Affiliations:** ^1^ Department of Biotechnology, Faculty of Agricultural Sciences, University of Guilan, Rasht, Iran; ^2^ Agricultural Biotechnology Research Institute of Iran (ABRII), North Region Branch, Agricultural Research, Education and Extension Organization (AREEO), Rasht, Iran; ^3^ Iranian Research Institute of Plant Protection, Agricultural Research, Education and Extension Organization (AREEO), Tehran, Iran

**Keywords:** bioinformatics, calcium homeostasis, codon usage bias, evolution, stress

## Abstract

Calcium (Ca^2+^) serves as a crucial intracellular messenger in plant signaling, particularly during stress responses. Precise regulation of calcium levels by transporters such as calcium/cation (CaCA) antiporters is essential for its effective function. However, the evolutionary dynamics and stress-related roles of the CaCA superfamily remain underexplored in key Brassicaceae crops. This study aimed to address this gap by investigating the hypothesis that *CaCA* genes in *Brassica napus*, *B. rapa*, and *B. oleracea* have undergone distinct evolutionary trajectories influencing their roles in abiotic stress responses, using Arabidopsis thaliana for comparison. Using Hidden Markov Model (HMM) profiling, 93 *CaCA* genes were identified across these species. These genes were categorized into four phylogenetic clades: CAX, CCX, NCL, and MHX. Comprehensive analyses of their coding proteins physicochemical properties, subcellular localization, conserved motifs, and gene structures were performed. Codon usage bias (CUB) analysis showed CaCA genes have low codon bias and CUB indices indicated a complex interplay between mutational and selective pressures, highlighting the influence of natural selection and mutational biases in shaping these genes. Collinearity and duplication analyses highlighted the evolutionary dynamics of the CaCA gene family, with several segmental and whole-genome duplication (WGD) events contributing to their expansion. Notably, duplicated genes underwent negative selection pressure, which removed harmful mutations, resulting in slower evolution and maintaining the functional stability of CaCA genes throughout their evolutionary history. Analysis of *cis-*regulatory elements (CREs) revealed their responsiveness to hormones and stresses, suggesting a potential role in plant environmental adaptation. Expression profiling of *CaCA* genes under abiotic stresses (dehydration, salinity, cold, and ABA) in *B. napus* was performed using publicly available RNA-seq datasets and analyzed with standard bioinformatics tools. Based on the results of expression analysis, key *CaCA* genes, such as *BnCAX3*, *BnCAX16*, *BnCC2*, *BnCCX9*, *BnCAX5*, *BnCAX12*, *BnCAX13*, and *BnMHX1*, which are differentially expressed and potentially crucial for stress tolerance. This comprehensive study elucidates the evolutionary architecture of the *CaCA* gene family in Brassicaceae and identifies key *BnCaCA* genes potentially crucial for abiotic stress tolerance, thus offering a foundation for future functional studies aimed at improving crop resilience.

## Introduction

A diverse array of cations, including copper (Cu^2+^), cobalt (Co^2+^), iron (Fe^2+^), magnesium (Mg^2+^), manganese (Mn^2+^), potassium (K^+^), nickel (Ni^2+^), and zinc (Zn) directly or indirectly contribute to plant biological processes at the cellular, organ, and whole-system levels. Among these essential elements, calcium (Ca^2+^) holds a pivotal role, serving as a crucial secondary messenger in plant signaling pathways during growth, development, and stress responses (Pilon et al., 2009). Calcium facilitates signal transduction in response to diverse internal and external stimuli (Dodd et al., 2010). Upon stimulation, plant cells experience a rise in calcium ion concentration, detected by calcium-binding or calcium-sensitive proteins. These proteins initiate a cascade of downstream signals, including phosphorylation events that ultimately regulate gene expression (Tuteja and Mahajan, 2007). The ubiquitous role of calcium signaling is evident in its response to growth regulators, nutrients, pathogens, and abiotic stresses, underscoring its importance in plant development and stress adaptation (Kader and Lindberg, 2010; Zhang et al., 2014).

The maintenance of Ca^2+^ homeostasis within plant cells is achieved through a sophisticated network of transporters and compartmentalization mechanisms. Among these, CaCA antiporters mediate the efflux of Ca^2+^ across the cell membrane through an antiport mechanism, exchanging cytosolic Ca^2+^ for monovalent cations such as H^+^, Na^+^, and K^+^, against their respective concentration gradients (Emery et al., 2012). The CaCA superfamily is composed of five primary families: YRBG, Na^+^/Ca^2^+ exchanger (NCX), Na^+^/Ca^2+^, K^+^ exchanger (NCKX), Cation/Ca^2^+ exchanger (CCX), and Cation/H^+^ exchanger (CAX) (Pittman and Hirschi, 2016). YRBG proteins are unique to prokaryotes, while CCX proteins are found only in eukaryotes. NCX and NCKX families occur in animals and algae but not in higher plants, whereas CAX proteins are present in a wide range of organisms, from bacteria to plants and animals.Additionally, two plant-specific CaCA groups have been identified: the EF-hand domain-containing CAX group (EF-hand CAX), also known as NCL and NCX-like proteins, and the Mg^2+^/H^+^ exchanger (MHX) group (Emery et al., 2012). The EF-hand CAX family, while distantly related to the CAX family, can exhibit Na^+^/Ca^2+^ activity similar to the NCX family (Li et al., 2016a) the MHX group has also been found to have an evolutionary relationship with the NCX family (Gaash et al., 2013).

Functional studies on *CaCA* genes reveal their essential roles in plant biology. In *Arabidopsis thaliana*, *CCX1* is up-regulated during leaf senescence, accelerating aging and modulating calcium signaling in response to ROS (Li et al., 2016b). *CCX2*, localized to the endoplasmic reticulum, is induced by salt and osmotic stress; its absence reduces stress tolerance and impairs growth due to disrupted calcium flux (Corso et al., 2018). In apple, *MdCCX1* and *MdCCX2* improve salt tolerance by reducing sodium and boosting antioxidant activity (Yang et al., 2021a, b). *CAX* genes are crucial for cation tolerance, metal transport, and stress responses: Arabidopsis *CAX1* mutants are hypersensitive to cadmium and oxidative stress (Baliardini et al., 2015; Ahmadi et al., 2018), while potato *StCAX1*/*4* and wheat *TaNCL2*-*A* enhance tolerance to cadmium, salt, and osmotic stress by promoting antioxidant defenses and supporting plant growth (Liu et al., 2023; Tyagi et al., 2023).

Despite extensive investigations into the CaCA superfamily in diverse plant species, including *Triticum aestivum* (Taneja et al., 2016), *Solanum lycopersicum* (Amagaya et al., 2019), *Oryza sativa*, *A. thaliana* (Pittman and Hirschi, 2016), *Malus domestica* (Mao et al., 2021), *Glycine max* (Zeng et al., 2020), *Zea mays* (Karami Lake et al., 2020), *Rosa roxburghii* (Zeng et al., 2024), and *Saccharum* sp*ontaneum* (Su et al., 2021), a comprehensive investigation into the evolutionary history, genomic organization, regulatory features, and specific contributions of the entire CaCA superfamily to abiotic stress responses in economically important Brassicaceae species like *Brassica napus*, *B. rapa*, and *B. oleracea* is currently lacking. This knowledge gap hinders targeted efforts to enhance stress tolerance in these crops. Therefore, the present study was undertaken to address these deficiencies. We hypothesized that the *CaCA* gene superfamily within these Brassicaceae species has undergone significant evolutionary diversification through gene duplication and selection, leading to members with specialized functions in abiotic stress adaptation. This work is anticipated to significantly advance our understanding of the evolutionary dynamics and functional significance of the *CaCA* gene superfamily in Brassicaceae. The findings are expected to provide valuable genomic resources and identify promising candidate genes for future functional validation and for developing strategies to improve abiotic stress resilience in these important crop species.

## Materials and methods

### Identification of the CaCA superfamily

The Hidden Markov Model (HMM) file for the Na_Ca_ex (sodium/calcium exchanger, PF01699) domain was retrieved from the Pfam database (Mistry et al., 2021) and used to search the proteomes of *B*. *napus*, *B. oleracea*, *B. rapa*, and *A. thaliana*, via the HMMsearch server with default parameters (Prakash et al., 2017) Identified sequences were further validated for The presence of the Na_Ca_ex domain using the SMART database. Sequences lacking the complete domain or exhibiting very short protein lengths were excluded from subsequent analyses (Letunic and Bork, 2018). All sequences utilized in this study—such as promoter, CDS, and protein sequences used for phylogenetic tree construction, along with the alignment file—are included in **Supplementary File 1**.

### Sequence characteristics and conserved motifs

The physicochemical properties of CaCA proteins, including protein length, molecular weight, and isoelectric point (pI), were calculated using the ProtParam server Subcellular localization predictions were performed using a combination of CELLO and ProtComp 9.0 servers (Yu et al., 2006). Transmembrane domains, critical for membrane-bound proteins like CaCAs, were identified using the DeepTMHHM server (Hallgren et al., 2022). Finally, conserved sequence motifs in CaCA protein sequences were identified using the Multiple Em for Motif Elicitation (MEME) suite (Bailey et al., 2015). The MEME server parameters were configured to detect a maximum of 10 motifs with lengths ranging from 6 to 100 amino acids.

### Sequence alignment and phylogenetic tree construction

To elucidate evolutionary relationships, full-length CaCA protein sequences from *T. aestivum*, *G. max*, *O. sativa*, *S. lycopersicum*, *A. thaliana*, *B. napus*, *B. oleracea*, and *B. rapa* were aligned using ClustalX v2.1 (Larkin et al., 2007). Subsequently, a phylogenetic tree was constructed from the aligned sequences using MEGA7 (Kumar et al., 2016) with The Maximum Likelihood (ML) algorithm and 1000 bootstrap replications. The resulting tree was visualized using iTOL v6 (Letunic and Bork, 2024). The selection of *B. napus*, *B. rapa*, and *B. oleracea* for this study was based on their close evolutionary relationship within the Brassicaceae family, their well-annotated and publicly available genomes, and their significant agricultural importance as major oilseed and vegetable crops*. A. thaliana* was included as a model organism and close relative, providing a reference point for comparative analysis within Brassicaceae. To assess the conservation and divergence of *CaCA* genes across angiosperms, we also incorporated representative species from other major plant lineages. These species were chosen for their phylogenetic diversity, availability of high-quality genome sequences, and relevance to global agriculture (**Supplementary File 1**).

### Gene structure analysis and promoter *cis*-regulatory elements identification

Gene structures of *CaCA* gene family members in *B. napus*, *B. rapa*, *B. oleracea*, and *A. thaliana* were analyzed using the GFF3 annotation files submitted to TBtools and information about gene structure and intron phases was obtained. TBtools was used to provide a graphical representation of gene structure and conserved motifs (Chen et al., 2020). Promoter regions, defined as the 1500 bp upstream of the start codon were analyzed for CREs using the PlantCARE server (Lescot et al., 2002).

### Codon usage bias

Codon usage bias (CUB) analysis was employed to investigate the patterns of codon usage in *CaCA* genes from *B. napus*, *B. rapa*, *B. oleracea*, and *A. thaliana*. The CodonW v1.4.2 software was utilized for this analysis. Various CUB indices were calculated, including Relative Synonymous Codon Usage (RSCU), Codon Bias Index (CBI), Frequency of Optimal Codons (FOP), Codon Adaptation Index (CAI), Effective Number of Codons (ENC), and GC content at the third codon position of synonymous codons (GC3s) (Bahiri-Elitzur and Tuller, 2021; Parvathy et al., 2022)To assess the relative contributions of mutation and natural selection in shaping the codon usage patterns of *CaCA* genes, neutrality plots were constructed. Neutrality plots compare the GC content at the first and second codon positions (GC12) to the GC (GC3) content at the third codon position (Sharp et al., 2010). PR2-plots further investigate the interplay between mutation and natural selection by evaluating the usage of A/T and G/C at the third codon position (Błażej et al., 2017; Chaudhary et al., 2022). ENC-plots provide another visual representation of the relative contributions of mutation and natural selection. If codon usage bias is mainly influenced by mutations, genes tend to fall along or close to the standard curve in the ENC-plot. Conversely, if natural selection plays a more significant role, genes tend to fall below the standard curve (Gao et al., 2022).

### Collinearity, duplication, and selection pressure analysis

Collinearity analysis was performed using MCScanX with default parameters (Wang et al., 2012). Duplication models were classified using the duplicated_gene_classifier tool. Collinearity relationships were visualized with the Advanced Circos tool in TBtools (Chen et al., 2020). The simple Ka/Ks calculator in TBtools software was used to determine the nonsynonymous substitution rate (Ka), synonymous substitution rate (Ks), and selection pressure (Ka/Ks) between duplicated *CaCA* genes (Chen et al., 2020). Ka/Ks < 1, Ka/Ks = 1, and Ka/Ks > 1 indicate negative, positive, and neutral selection pressure, respectively (Abedi et al., 2021).

### Gene expression analysis of *CaCA* genes in *B. napus* under abiotic stresses

To investigate the expression patterns of *BnCaCA* genes under abiotic stress conditions, we analyzed RNA-seq datasets from the National Genomics Data Center (NGDC) under project ID CRA001775. These datasets included samples subjected to dehydration, salinity (200 mM), cold (4°C), and ABA (25 µM) treatments. The experiment included three biological replicates. Sampling For dehydration stress occurred at 1 and 8 hours post-treatment, while for salt, cold, and ABA stresses, sampling was performed at 4 and 24 hours post-treatment.

Quality control and preprocessing of raw reads were performed using FastQC and Trimmomatic, respectively (Andrews et al., 2010; Bolger et al., 2014). High-quality reads were aligned to the *B. napus* genome using the STAR software (Dobin et al., 2013). Differentially expressed genes (DEGs) were identified using the DEseq2 package with criteria of |log2 (fold change)| >1 and adjusted p-value <0.01 for significant expression changes (Love et al., 2014).

### Software availability

Pfam: http://pfam.xfam.org/; HMMsearch: https://www.ebi.ac.uk/Tools/hmmer/search/hmmsearch; SMART: http://smart.embl-heidelberg.de/; ProtParam: https://web.expasy.org/protparam/; CELLO: http://cello.life.nctu.edu.tw/; ProtComp 9.0: http://www.softberry.com/; DeepTMHHM: https://dtu.biolib.com/DeepTMHMM; Multiple Em for Motif Elicitation: https://meme-suite.org/meme/tools/meme; ClustalX v2.1: https://clustalx.software.informer.com/; MEGA7: https://www.megasoftware.net/; iTOL v6: https://itol.embl.de/; PlantCARE: https://bioinformatics.psb.ugent.be/webtools/plantcare/html/; CodonW v1.4.2: software https://sourceforge.net/projects/codonw/; MCScanX: https://github.com/wyp1125/MCScanX; NGDC: https://ngdc.cncb.ac.cn/?lang=en; TBtools: https://github.com/CJ-Chen/TBtools/releases; FastQC: https://www.bioinformatics.babraham.ac.uk/projects/fastqc/; Trimmomatic: http://www.usadellab.org/cms/?page=trimmomatic; STAR: http://code.google.com/p/rna-star/; DEseq2: http://www.bioconductor.org/packages/release/bioc/html/DESeq2.html.

## Results and discussion

### CaCA superfamily in B. napus, B. rapa, B. oleracea, and A. thaliana

The genome of *B. napus*, *B. rapa*, *B. oleracea*, and *A. thaliana* was searched using the HMM profile of the Na_Ca_ex domain, revealing 40 genes encoding BnCaCA (17 CAX, 15 CCX, 2 MHX, 6 NCL) in *B. napus*, 20 genes encoding BoCaCA (8 CAX, 8 CCX, 1 MHX, 3 NCL) in *B. oleracea*, 19 genes encoding BrCaCA (8 CAX, 7 CCX, 1 MHX, 3 NCL) in *B. rapa*, and 14 genes encoding AtCaCA (6 CAX, 5 CCX, 1 MHX, 2 NCL) in *A. thaliana* (**Supplementary File 2**). Analysis of the physicochemical properties of the identified proteins showed that the average molecular weight and length of CaCA proteins for *B. napus* are 55.54 kDa and 511.35 amino acids, for *B. oleracea* are 55.47 kDa and 509.15 amino acids, for *B. rapa* are 57.2 kDa and 523 amino acids, and for *A. thaliana* are 57.72 kDa and 526.78 amino acids. The BnNCL2, with a molecular weight of 71.43 kDa and a length of 649 amino acids, has the highest molecular weight and length, while the lowest molecular weight and length are found in BnCCX10 and BoCCX3, which have 347 amino acids and a molecular weight of 37.02 kDa. The isoelectric point of CaCA proteins in the studied plants falls within the acidic to alkaline range. The average pI for *B. napus*, *B. oleracea*, *B. rapa*, and *A. thaliana* is 6, 5.87, 6.03, and 5.89, respectively. The lowest pI is for *BoCAX1* at 4.7, and the highest is for *BrCAX7* at 9.22 (**Supplementary File 2**). The study of transmembrane domains showed that, except for BnCAX17 and BnCAX7, which have eight transmembrane domains, other proteins in this group have 11 transmembrane domains (**Supplementary File 2**). Additionally, except for BnNCL2, which has 11 transmembrane domains, other members of the NCL and MHX subgroups have 10 transmembrane domains (**Supplementary File 2**). In the CCX subgroup, although most members have 13 transmembrane domains, some have fewer. For example, BnCCX10, BoCCX3, and BrCCX1 have eight transmembrane domains (**Supplementary File 2**). Predicting the cellular localization of CaCA proteins using the CELLO server showed that these proteins can be present in the vacuole, plasma membrane, lysosome, endoplasmic reticulum, and Golgi apparatus. On the other hand, the ProtComp 9.0 server predicted the intracellular localization of NCL subgroup proteins to be extracellular, the localization of BnCCX5, BnCCX13, BoCCX6, and AtCAX5 to be plasma membrane-bound, and other CaCA proteins to be membrane-bound vacuolar. The prediction of the localization of CaCA proteins in *S. lycopersicum* and *T. aestivum* also showed that these proteins are generally present in the vacuole (Taneja et al., 2016; Amagaya et al., 2019). The results of the ProtComp 9.0 server are largely consistent with experimental studies on the localization of CaCA proteins. For example, it has been shown that AtCAX1, AtCAX2, AtMHX, AtCCX3, and OsCCX2 are present in the vacuole membrane (Shaul et al., 1999; Hirschi et al., 2000; Kamiya et al., 2006; Morris et al., 2008; Yadav et al., 2015). On the other hand, ScCAX4 can be present in the nucleus, plasma membrane, and cytoplasm, while *RrCAX1a* is localized to the cell membrane (Su et al., 2021; Zeng et al., 2024).

### Phylogenetic analysis of the CaCA superfamily

**Figure 1:**
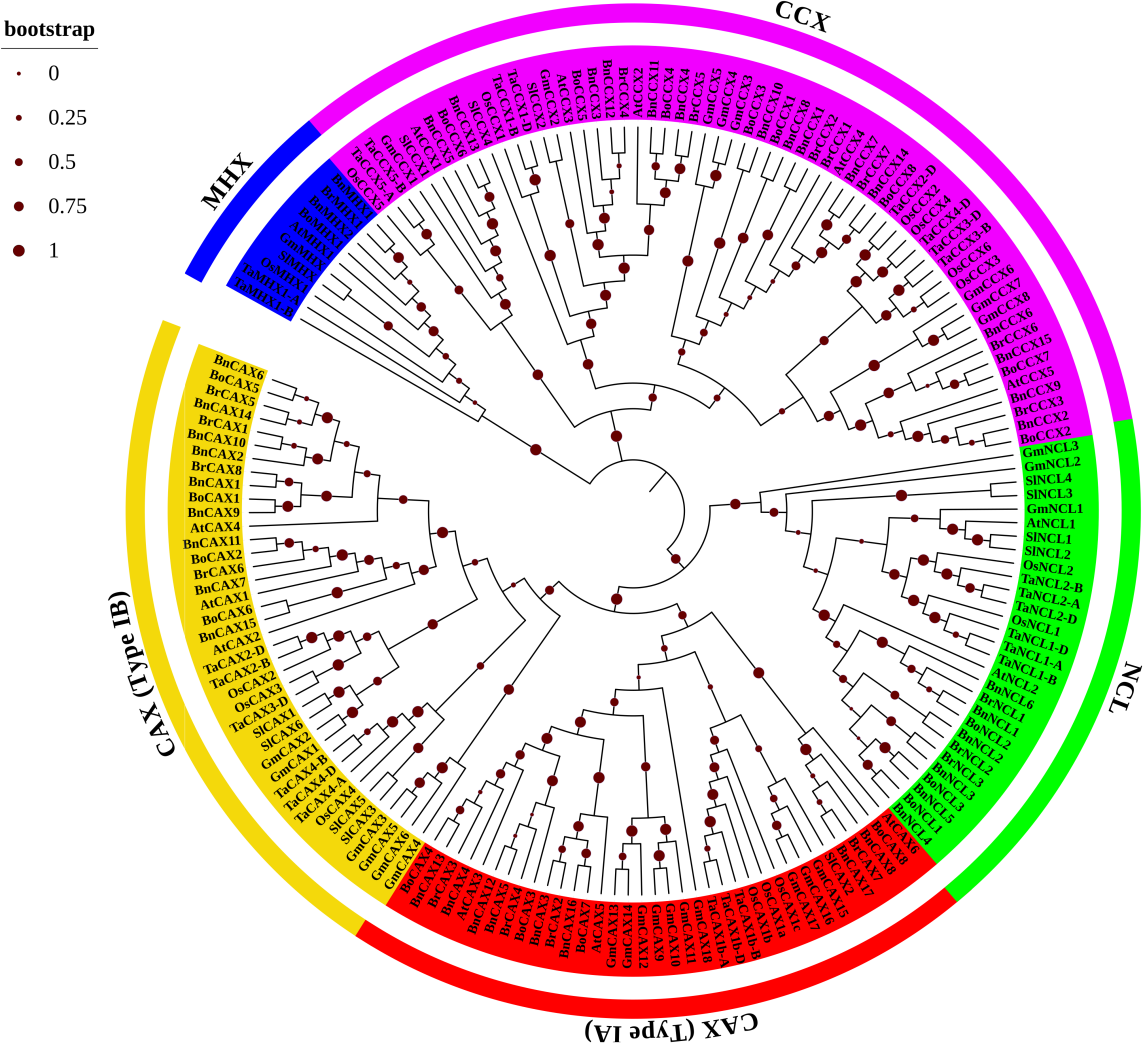
Phylogenetic tree of CaCA proteins from *B*. *napus* (Bn), *B*. *oleracea* (Bo), *B*. *rapa* (Br), *A*. *thaliana* (At), *O. sativa* (Os), *T. aestivum* (Ta), *G*. *max* (Gm), and *S. lycopersicum* (Sl). The phylogenetic tree was constructed using the Maximum Likelihood method based on full-length CaCA protein sequences. Four main groups are indicated: CAX (red and yellow branches), CCX (pink branches), NCL (green branches), and MHX (blue branches). Red circles indicate bootstrap values.

To elucidate the evolutionary relationships among plant *CaCA* genes, eight plant species were selected for analysis: *A. thaliana* (At), *B. napus* (Bn), *B. rapa* (Br), *B. oleracea* (Bo), *G. max* (Gm), *S. lycopersicum* (Sl), *T. aestivum* (Ta), and *O. sativa* (Os). A total of 147 CaCA protein sequences from these monocot and dicot species were categorized into four phylogenetic groups based on sequence similarity: CAX, NCL, MHX, and CCX (**Figure 1**).

The phylogenetic analysis revealed a close evolutionary relationship between the NCL and CAX groups. This finding suggests that, despite their functional differences, these two groups may share a common ancestral origin and have retained certain conserved features throughout evolution. The close clustering of NCL and CAX proteins in the phylogenetic tree provides new insights into the evolutionary dynamics of the CaCA superfamily in Brassicaceae. Similarly, the MHX and CCX groups clustered together, consistent with previous reports (Emery et al., 2012; Pittman and Hirschi, 2016; Taneja et al., 2016). Within each group, genes from dicot plants clustered more closely, reflecting their shared evolutionary history. The highest degree of relatedness was observed among *A. thaliana*, *B. napus*, *B. oleracea*, and *B. rapa*, all belonging to the Brassicaceae family (**Figure 1**). Additionally, a close evolutionary relationship is observed between the genes of *T. aestivum* and *O. sativa* because both species belong to the Poaceae family. The analysis identified the CAX (75 members) and CCX (60 members) groups as the largest, followed by the NCL (29 members) and MHX (10 members) groups (**Figure 1**). Notably, the CAX group can be further subdivided into groups I-A and I-B (Emery et al., 2012; Amagaya et al., 2019). Previous studies suggest that group I-A is specific to both monocots and dicots, while group I-B includes mosses in addition to these groups. This pattern suggests evolutionary divergence within the CAX family, potentially contributing to functional differences between these subgroups (Emery et al., 2012; Taneja et al., 2016; Su et al., 2021).

### Gene structure and intron phase

**Figure 2:**
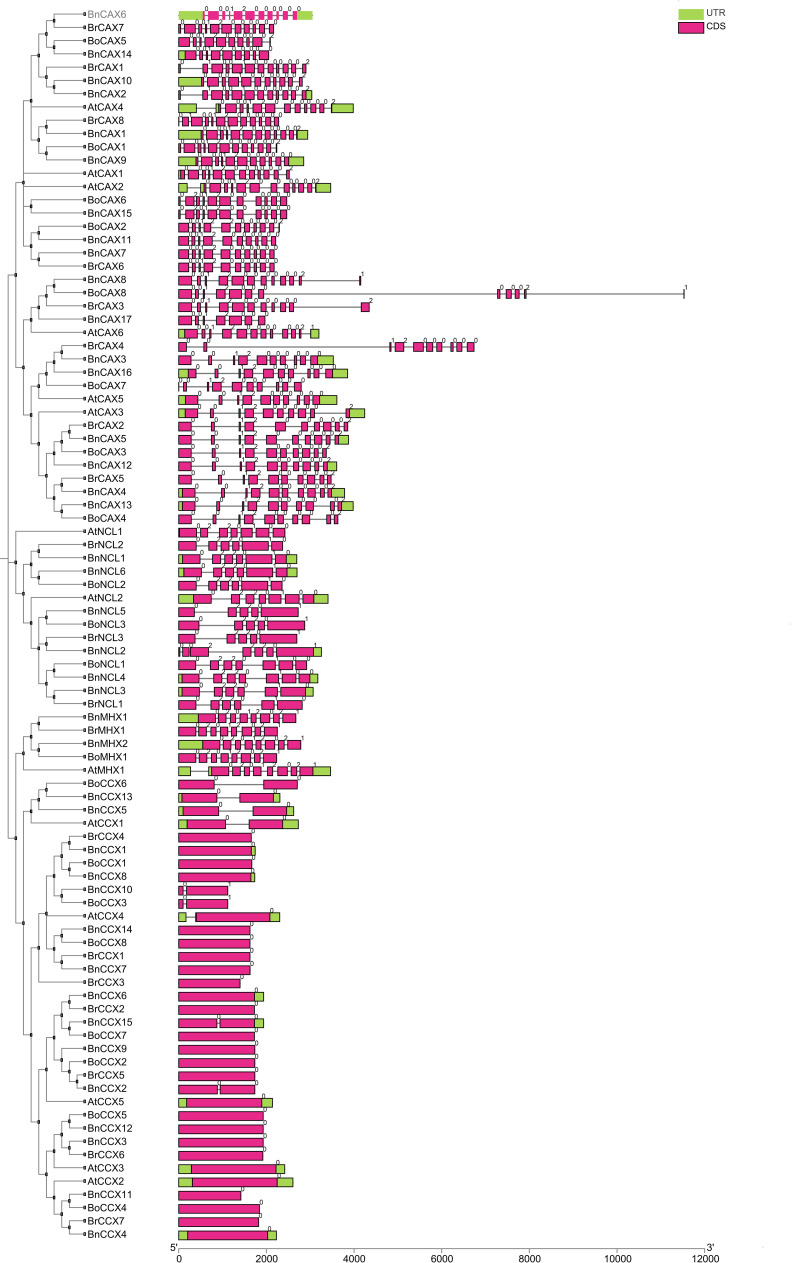
Gene structure of *CaCA* genes in *B*. *napus*, *B*. *oleracea*, *B*. *rapa*, and *A*. *thaliana*. For each gene, the corresponding gene structure is displayed to the right, with coding sequences (CDS) shown as magenta boxes and untranslated regions (UTRs) shown as green boxes. Black lines represent introns. Gene names are labeled according to species: Bn (*B. napus*), Br (*B. rapa*), Bo (*B. oleracea*), and At (*A. thaliana*). The arrangement of exons and introns is drawn to scale, as indicated by the scale bar at the bottom. This visualization allows comparison of exon-intron organization among *CaCA* gene family members and across different species.

Analysis of gene structure is crucial for understanding gene function, organization, and evolution (Hu et al., 2024). The exon-intron structure of *CaCA* genes in *A. thaliana*, *B. napus*, *B. rapa*, and *B. oleracea* revealed distinct patterns across subgroups (**Figure 2**). The CCX subgroup genes have a maximum of one intron, while The CAX subgroup genes contain 8–11 introns, the NCL subgroup genes have 4–6 introns, and the MHX subgroup genes contain 7 introns. Eukaryotic genes are classified into three groups based on the number of introns: intronless, intron-poor (three or fewer introns), and intron-rich (Liu et al., 2021). Accordingly, the CCX subgroup genes are intron-poor, whereas genes in the other subgroups are intron-rich. The analysis also showed that each *CaCA* subgroup has its unique intron pattern. For example, *BoCAX8* and *BrCAX4* genes, due to their long introns, are the longest genes in this study (**Figure 2**).

Gene structure analysis of the CaCA superfamily in *M. domestica*, *T. aestivum*, and *S. lycopersicum* revealed similar patterns. Specifically, CCX subgroup genes in these plants have zero or one intron, while the other subgroups are intron-rich. In *M. domestica*, MHX subgroup genes contain eight introns, NCL subgroup genes have six introns, and CAX subgroup genes contain 10 to 12 introns. In *S. lycopersicum* and *T. aestivum*, MHX subgroup genes have seven introns, while CAX subgroup genes in *S. lycopersicum* have 6 to 11 introns and in *M. domestica*, they range from 10 to 12 introns. The NCL subgroup of *S. lycopersicum* features 6 to 8 introns, while in *M. domestica*, it consistently has six introns (Taneja et al., 2016; Amagaya et al., 2019; Mao et al., 2021). Hence, in these plants, strong conservation is maintained within each subgroup, but substantial sequence and structural variations exist among the different subgroups (Zeng et al., 2024). Evolutionarily, genes with few or no introns are considered part of a plant’s adaptation strategy for rapid responses to biotic and abiotic stresses. Thus, CCX subgroup genes may play a significant role in stress response (Liu et al., 2021).

Introns are classified into three types based on their phase: zero, one, and two. In phase zero, the intron is located between two codons; in phase one, it is situated between the first and second nucleotides of a codon; and in phase two, the intron is positioned between the second and third nucleotides of a codon (Wu et al., 2023). An analysis of intron phases revealed the highest diversity in the CAX, NCL, CCX, and MHX subgroups. Twelve intron phase patterns, including 00012000000, 0012000002, 001200000, 010010000002, 00010000002, 000120000002, 010012000000, 02012000000, 00122000002, 0012000000, 001200000021, and 0012000, were observed in the CAX subgroup (**Figure 2**). The CCX subgroup displayed the least intron phase variation, with only two patterns (0 and 01). Phase 0 dominated in this subgroup (95%), with phase one being a minor component (5%). Notably, phase two introns were absent in the CCX subgroup (**Figure 2**). The MHX subgroup exhibited the most uniform intron phase pattern, with all genes sharing a single pattern (02012021). Here, phases zero and two occurred with equal frequency (37.5%), while phase one was observed less frequently (10%) (**Figure 2**). across all *CaCA* subgroups, phase 0 introns consistently displayed the highest frequency, followed by phases one and two, respectively (Nguyen et al., 2006). The conservation levels of intron phases closely matched their frequencies, with phases zero, one, and two showing the highest levels of conservation (Long and Deutsch, 1999). The frequency of phase zero introns was highest across all *CaCA* subgroups, indicating that the gene structure and intron phase patterns are highly conserved. Similar findings were observed in the *TaCaCA* genes of *T. aestivum*, where The frequencies of intron phases zero, one, and two were 62%, 23%, and 14%, respectively (Taneja et al., 2016).

### Conserved motifs

**Figure 3:**
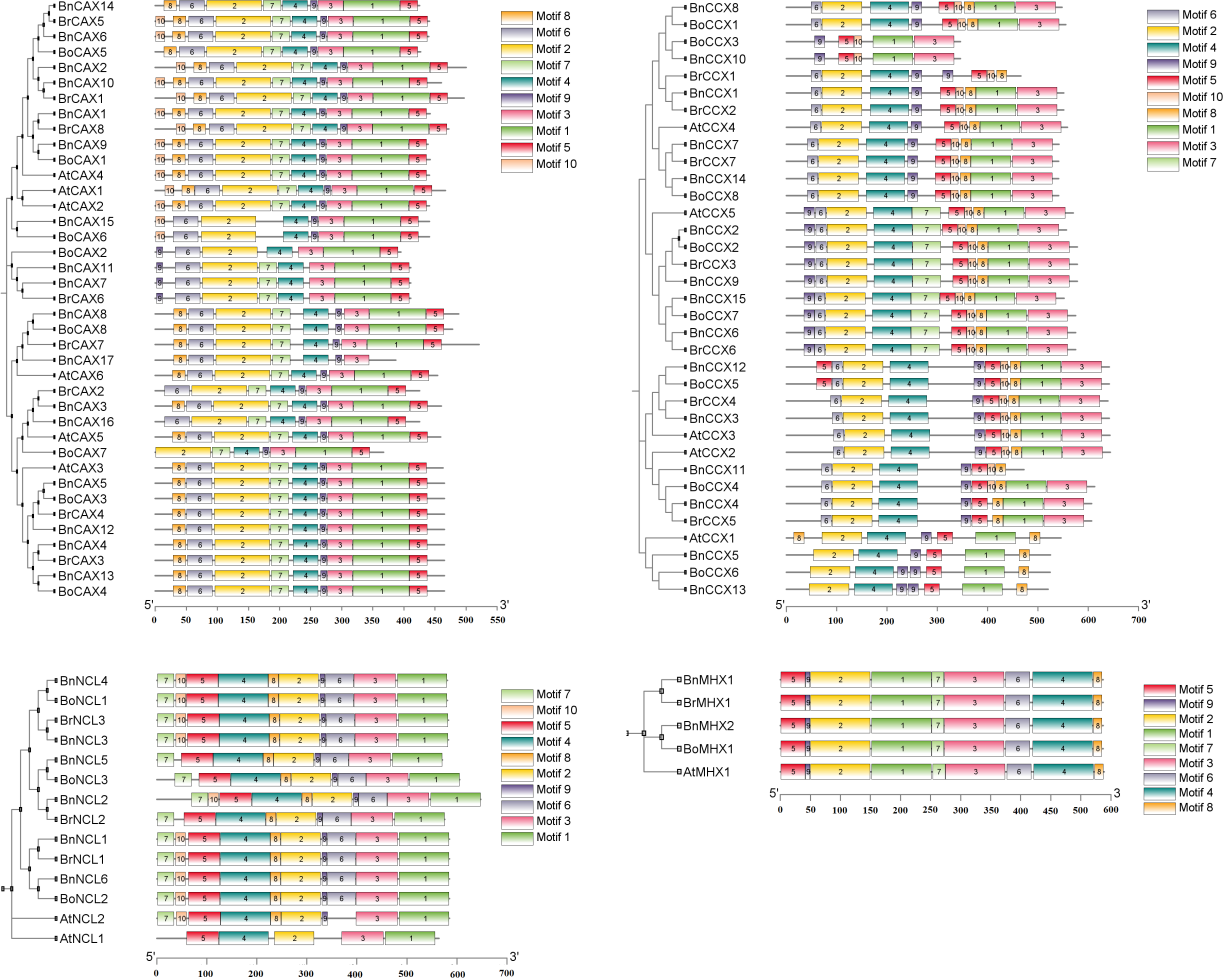
Conserved motif analysis of CaCA superfamily members in *B*. *napus*, *B*. *oleracea*, *B*. *rapa*, and *A*. *thaliana*. The distribution of conserved motifs identified by MEME is shown for four subgroups of the CaCA superfamily: CAX (top left), CCX (top right), NCL (bottom left), and MHX (bottom right). Each colored box represents a distinct conserved motif. Motif positions within each protein sequence are indicated by the scale at the bottom.

Based on the analysis, 10 conserved motifs were identified in the CAX, CCX, and NCL subgroups, while nine conserved motifs were found in the MHX subgroup. Variations in the abundance, length, and function of these motifs were observed in the different subgroups (**Figure 3**, **Supplementary File 2**). In the CAX subgroup, the motifs ranged from 11 to 91 amino acids in length, with motifs 1, 2, and 4 associated with the Na_Ca_ex (PF01699) domain. In the CCX subgroup, motif lengths varied between 15 and 80 amino acids, and motifs 1 through 6 belonged to the Na_Ca_ex domain (**Figure 3**, **Supplementary Table 2**). The MHX subgroup contained nine motifs ranging from 8 to 100 amino acids, among which motifs 1, 2, and 4 exhibited Na_Ca_ex functionality (**Figure 3**, **Supplementary Table 2**). It can generally be said that the CCX (excluding *BoCCX3*, *BnCCX10*, and *BrCCX1*), CAX, and MHX subgroups each possess two Na-Ca-ex domains located in both the N-terminal and C-terminal halves (Taneja et al., 2016; Su et al., 2021). Unlike the other subgroups, the NCL subgroup featured motif 2 with EF-hand function in addition to motif 1, which is part of the Na_Ca_ex domain. The motifs in NCL ranged in length from 11 to 100 amino acids (**Figure 3**, **Supplementary Table 2**).

Previous studies have shown that these proteins contain two conserved regions called α-repeats, which play key roles in ion selectivity, binding, and transport (Emery et al., 2012; Gaash et al., 2013; Taneja et al., 2016). In Brassicaceae species, analysis of proteins from this family revealed that in the CAX subgroup, the α1-repeat and α2-repeat regions are located in conserved motifs 2 and 1, respectively (**Supplementary File 3**). A distinctive feature of the α-repeats in this subgroup is the presence of the signature motif “GNxxE” where glutamate (E) residues play a crucial role in ion transport (**Supplementary File 3**) (Kamiya and Maeshima, 2004; Waight et al., 2013; Mao et al., 2021). Glycine (G) residues within these regions have also been shown to contribute to the conformational flexibility of these proteins (Shigaki et al., 2006). In the CCX subgroup, the α1-repeat region is found in conserved motifs 2 and 4, while the α2-repeat region is located in conserved motifs 1 and 3. The signature motifs “GNGAPD” in the α1-repeat and “GNSxGD” in the α2-repeat are conserved. Additionally, the motifs “A(G/A)VTLL” in the α1-repeat and “L(G/A)xTVALAW” in the α2-repeat are observed (**Supplementary File 3**) (Emery et al., 2012). The MHX subgroup has the α1-repeat region identified in conserved motif 2, with the signature motif “GTSFPQ”. The α2-repeat region is located in conserved motifs 4 and 6, featuring the signature motif “GTSWPD” (**Supplementary File 3**) (Taneja et al., 2016). It appears that the amino acid residues in the protein’s α1 region play a crucial role in ion recognition and selectivity, particularly in the exchange of Mg^2+^ and Zn^2+^/H^+^ ions (Shigekawa et al., 2002; Ottolia et al., 2005; Emery et al., 2012). The NCL subgroup contains only one α-repeat region and, as previously mentioned, also possesses an EF-hand domain involved in calcium binding (**Supplementary File 3**). It appears that negatively charged or acidic amino acid residues such as glutamate (E), aspartate (D), and serine (S) in this domain play an important role in electrostatic interactions with positively charged Ca^2+^ ions (Ikura, 1996; Taneja et al., 2016). These findings suggest that each subgroup of the CaCA superfamily in *A. thaliana*, *B. napus*, *B. rapa*, and *B. oleracea* differs in gene structure, intron phase, and conserved motifs, consistent with their phylogenetic relationships and functional divergence.

### Codon usage bias

**Figure 4:**
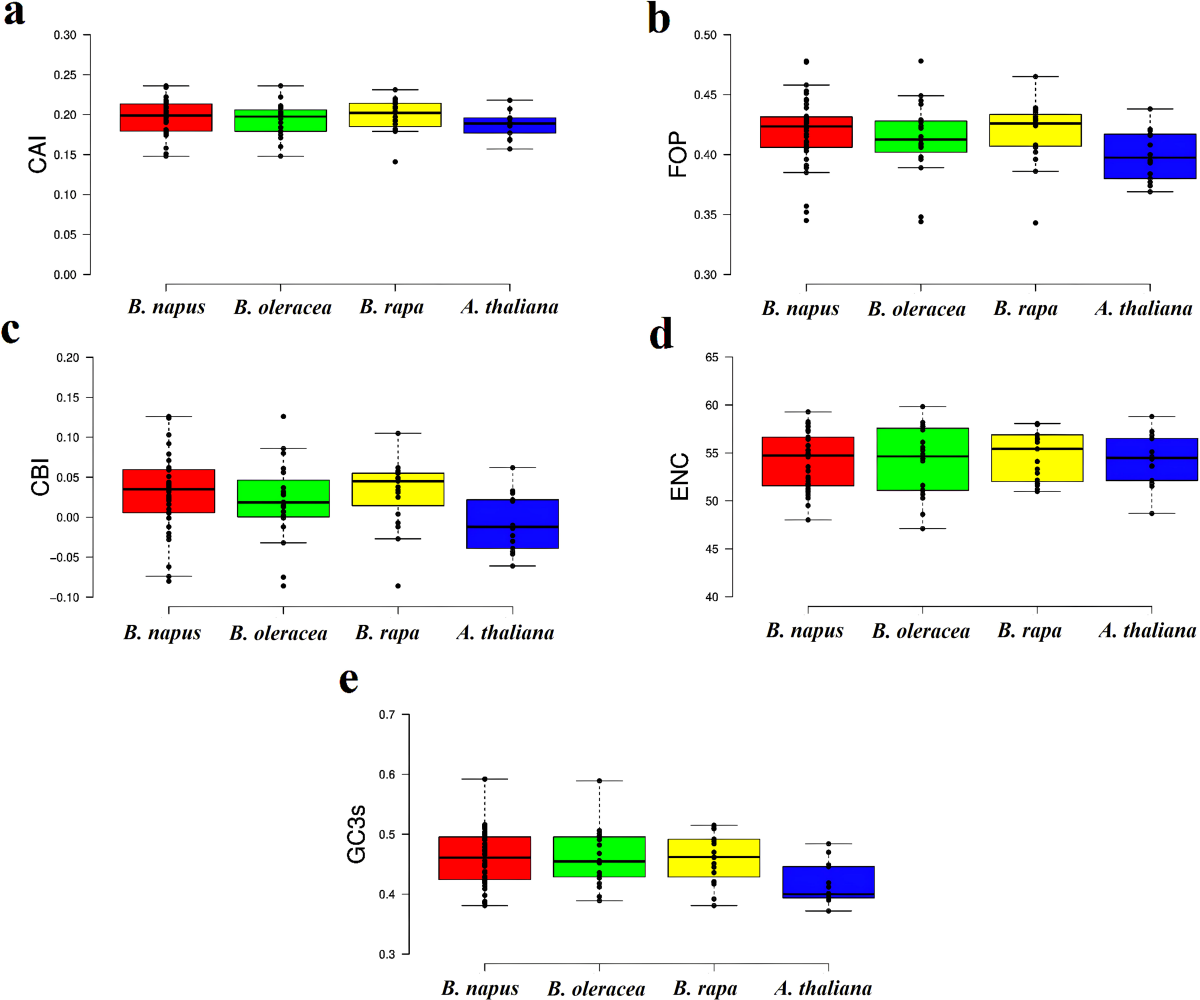
Box and whisker plots of codon bias parameters for the CaCA superfamily in *B. napus, B. oleracea, B. rapa*, and *A. thaliana*. The indices are shown as follows: **(a)** CAI, **(b)** FOP, **(c)** CBI, **(d)** ENC, and **(e)** GC3s. In each graph, the values for *B. napus*, *B. oleracea*, *B. rapa*, and *A. thaliana* are shown in red, green, yellow, and blue boxes, respectively.

Codon usage bias (CUB) is a widespread phenomenon in gene families, where certain synonymous codons are preferentially used over others to encode the same amino acid. This non-random codon usage has significant implications for understanding gene expression regulation, evolutionary relationships, and adaptation in plants (Parvathy et al., 2022). To investigate this phenomenon, we calculated several codon bias indices (CAI, FOP, ENC, CBI, GC3s, and RSCU) for *CaCA* genes in *B. napus*, *B. rapa*, *B. oleracea*, and *A. thaliana*. The results indicated that the CAI values for *CaCA* genes ranged from 0.14 to 0.23, with a mean of 0.195. The FOP values ranged from 0.34 to 0.43, with a mean of 0.41. The CBI values of the *CaCA* genes ranged from -0.08 to 0.12, with a mean of 0.02. ENC analysis showed that the ENC values ranged from 47.11 to 58.07, with a mean of 54.3. The GC3 values ranged from 0.3 to 0.48, with a mean of 0.45 (**Figure 4**, **Supplementary File 5**). Based on the CAI, FOP, ENC, GC3s, and CBI indices, it can be inferred that *CaCA* genes in *B. napus*, *B. rapa*, *B. oleracea*, and *A. thaliana* exhibit weak codon usage bias. Additionally, the CAI and FOP values suggest low expression levels (Eshkiki et al., 2020). This weak codon usage bias in the *CaCA* genes may result from relaxed selection pressures or a balance between mutation and selection (Hershberg and Petrov, 2008; Gao et al., 2022). The analysis also revealed that 22 codons had an RSCU value greater than one, with seven codons (TTC, TTG, ATC, GTC, TAC, AAC, and AGG) ending in C/G and 15 codons (CTT, GTT, TCT, CCT, CCA, ACT, ACA, GCT, CAT, CAA, GAT, TGT, AGA, GGT, and GGA) ending in A/T (**Supplementary File 6**). These findings suggest that codons ending in A/T are preferentially used, which aligns with previous studies indicating that dicot plants tend to prefer codons ending in A/T (Kawabe and Miyashita, 2003).

**Figure 5:**
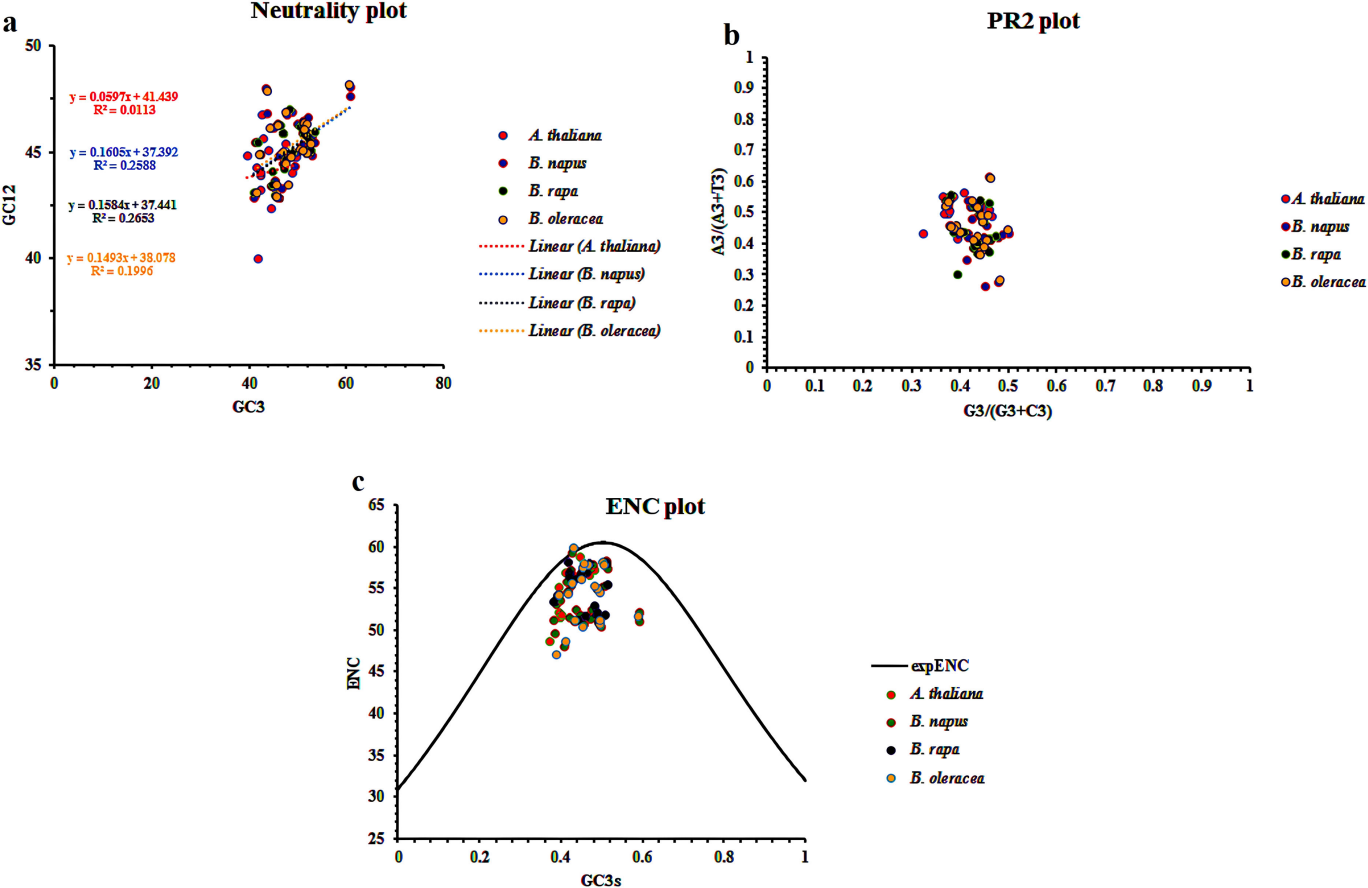
Neutrality plot **(a)**, PR2-plot **(b)**, and ENC-plot **(c)** analysis of the CaCA superfamily in *B*. *napus*, *B*. *oleracea*, *B*. *rapa*, and *A*. *thaliana*. In all three plots, each point represents a gene, and different colors are used to indicate the genes from each plant species.

To understand how mutation and natural selection influence codon usage patterns within the CaCA superfamily across *B. napus*, *B. rapa*, *B. oleracea*, and *A. thaliana*, we employed Neutrality Plots, PR2 plots, and ENC plots (Chaudhary et al., 2022). The Neutrality Plot analysis revealed r values of 0.1, 0.5, 0.43, and 0.5 for *CaCA* genes in *A. thaliana*, *B. napus*, *B. oleracea*, and *B. rapa*, respectively. The slopes of the regression lines for these genes were 0.059 (*A. thaliana*), 0.16 (*B. napus*), 0.149 (*B. oleracea*), and 0.158 (*B. rapa*) (**Figure 5a**). Interpreting these results suggests that natural selection primarily influences codon usage patterns in *A. thaliana*. However, in the other three species, while natural selection remains the dominant force, mutation also contributes to shaping codon usage patterns.

PR2 plot results revealed that for *A. thaliana*, most genes were positioned at the top of the horizontal axis, while for *B. napus*, *B. rapa*, and *B. oleracea*, they were located at the bottom. Regarding the vertical axis, *CaCA* genes in all four plants are clustered on the left side (**Figure 5b**). These results indicate that in *A. thaliana*, the usage of A and G bases is more prominent than T and C bases at three positions, while for the other three plants, T and C bases are more frequently used. Thus, natural selection primarily influences the codon usage bias (CUB) of *AtCaCAs*, *BrCaCAs*, *BnCaCAs*, and *BoCaCAs* genes.

Further analysis using the ENC plot revealed that the distribution of *CaCA* genes in all four plants fell below the standard curve, suggesting that the influence of natural selection on CUB is greater than that of mutation (**Figure 5c**). Therefore, it can be concluded that both natural selection and mutation contribute to shaping codon usage bias, as indicated by the results from CUB indices such as FOP, CBI, ENC, and CAI. Studies on other gene families, such as *Catalase* (*CAT*), *Autophagy-Related Genes* (*ATG*), and *Fatty Acid Desaturase 2* (*FAD2*) in *Brassica* plants, also show the simultaneous influence of natural selection and mutation on codon usage (Eshkiki et al., 2020; Chaudhary et al., 2022; Sarcheshmeh et al., 2023). These findings highlight the dynamic interplay between mutation and natural selection in shaping the genome and transcriptome of plants. Furthermore, the observed differences between *A. thaliana* and *Brassica* species likely reflect their distinct evolutionary histories and ecological adaptations.

### Chromosomal location, duplication, selection pressure, and collinearity

**Figure 6:**
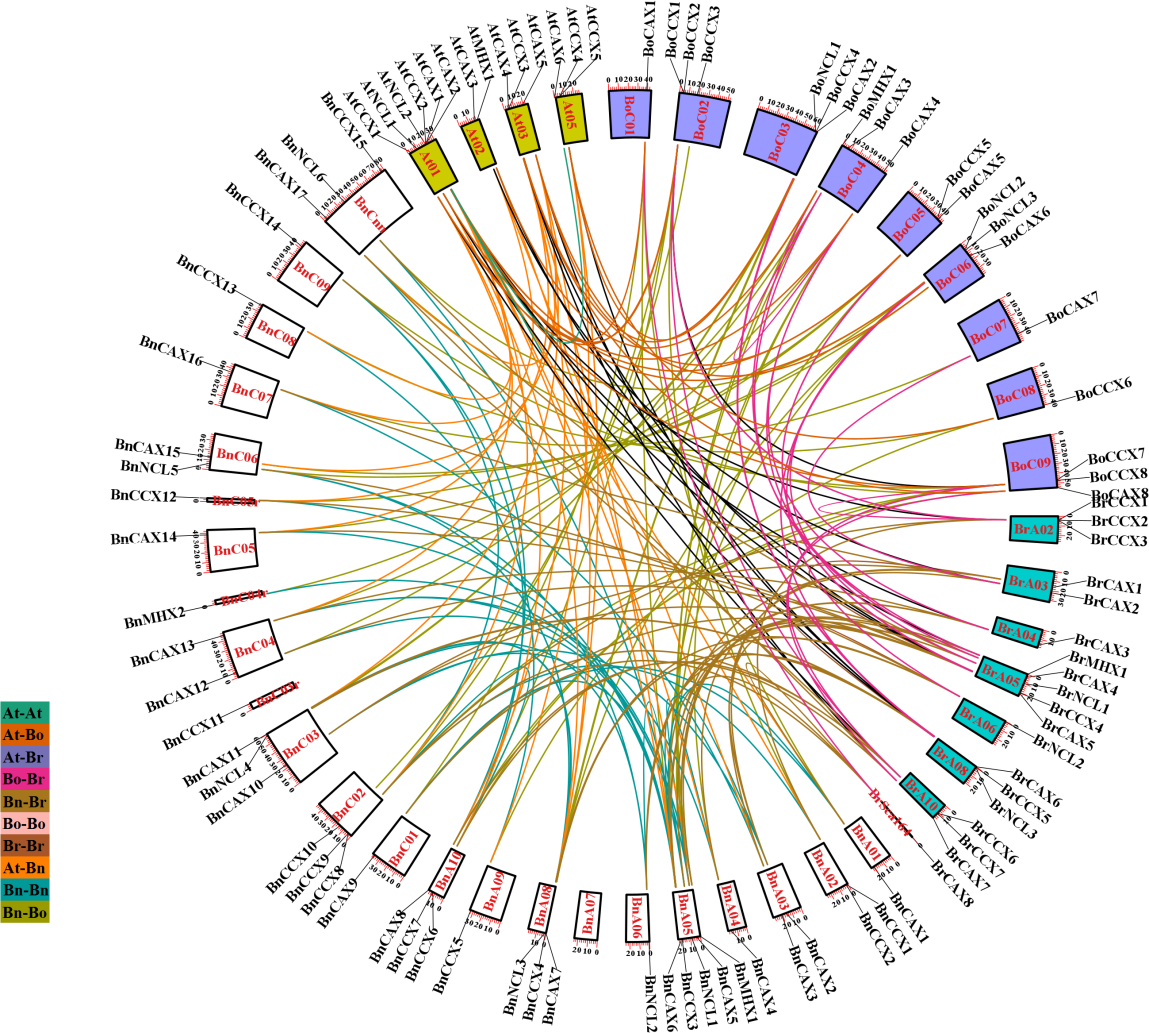
Intraspecies and interspecies synteny analysis of the CaCA superfamily in *B*. *napus*, *B*. *oleracea*, *B*. *rapa*, and *A*. *thaliana*. Chromosomes of *B*. *napus* are shown in black, *B*. *rapa* in light gray, *B*. *oleracea* in dark gray, and *A*. *thaliana* in white. Syntenic *CaCA* genes are connected by curved lines in various colors.

The localization of *BnCaCA* genes indicates that these genes are unevenly distributed across the chromosomes. The A and C subgenomes of *B. napus* contain 19 and 18 *CaCA* genes, respectively. Additionally, *BnCAX17*, *BnCCX15*, and *BnNCL6* are located on random chromosomes, which prevents precise localization. The results show that, except for chromosome BnA07, all other chromosomes of *B. napus* contain at least one gene from the CaCA superfamily. In *A. thaliana*, *B. rapa*, and *B. oleracea*, *CaCA* genes are also unevenly distributed across chromosomes. Notably, chromosomes Br01, Br07, and Br07 of *B. rapa* and chromosome At04 of *A. thaliana* lack *CaCA* genes (**Figure 6**).

Genomic dynamism facilitates the generation of genetic novelty, a prerequisite for species evolution and adaptation to changing environments. Gene duplication represents a crucial mechanism for introducing new genetic material and facilitating the acquisition of novel functions (Lallemand et al., 2020). To investigate the role of duplication events in the expansion of *CaCA* genes, we employed the MCScanX software (Wang et al., 2012), which identifies five distinct duplication types: singleton, dispersed, proximal, tandem, and whole-genome duplication (WGD) or segmental duplication. The analysis revealed that segmental/WGD duplication has been the primary driver of *CaCA* gene expansion in these plants, except *BnCCX11* and *BnCCX15*, which originated through dispersed duplication (**Figure 6**). This suggests that segmental duplications/WGD have played a significant role in shaping the CaCA superfamily within these species.

To elucidate the evolutionary trajectory of the CaCA superfamily in the Brassicaceae family, we conducted collinearity analysis among *A. thaliana*, *B*. *napus*, B. *rapa*, and *B. oleracea* to identify orthologous and paralogous *CaCA* genes. Paralogous genes arise through duplication events, while orthologous genes diverge during speciation (Stamboulian et al., 2020). Our analysis revealed 28 *CaCA* paralogous gene pairs in *B. napus*, two each in *B. rapa* and *B. oleracea*, and one in *A. thaliana* (**Figure 6**, **Supplementary File 7**). Additionally, 154 gene pairs exhibited orthologous relationships, distributed as follows: 23 At-Bn, 12 At-Br, 15 At-Bo, 39 Bn-Bo, 44 Bn-Br, and 21 Br-Bo (**Figure 6**, **Supplementary File 7**). Interestingly, the *B. napus* genes *BnCCX11* and *BnCCX15* lacked collinearity with other genes, suggesting their origin through dispersed duplication events. Studies on this gene family in *S.* sp*ontaneum* and *M. domestica* further support the idea that segmental duplication/WGD is a major driver of its expansion (Mao et al., 2021; Su et al., 2021).

To investigate the selection pressure acting on duplicated genes, we calculated Ka, Ks, and Ka/Ks values for both paralogous and orthologous gene pairs. The analysis revealed that most duplicated gene pairs (excluding 18 for which Ka/Ks could not be determined) experienced negative selection pressure during evolution (average Ka/Ks = 0.15) (**Supplementary File 7**). Negative selection pressure removes deleterious mutations from the gene, leading to a slower rate of evolution and resulting in a more stable gene structure and function (Zeng et al., 2024). Therefore, we infer that the *CaCA* genes in the studied plants have exhibited functional conservation throughout their evolutionary history.

Evolutionary studies suggest that the divergence between *Brassica* and *Arabidopsis* lineages occurred approximately 20–40 million years ago, followed by a Whole Genome Triplication (WGT) event, which gave rise to the *Brassica* species *B. rapa* and *B. oleracea* (Beilstein et al., 2010; Wei et al., 2023) Notably, *B. napus* emerged through the natural hybridization of *B. rapa* and *B. oleracea* around 7,500 years ago (Beilstein et al., 2010). Based on these evolutionary events, it would be expected that for every gene present in *A. thaliana*, there should be three copies in the *B. oleracea* and *B. rapa* genomes, and six copies in the *B. napus* genome. Since *A. thaliana* possesses 14 *CaCA* genes, the theoretical numbers for *B. rapa*, *B. oleracea*, and *B. napus* would be 42, 42, and 84 *CaCA* genes, respectively. However, the actual number of *CaCA* genes identified in these *Brassica* species deviates significantly from the predicted values. Our analysis revealed that *B. oleracea*, *B. rapa*, and *B. napus* have lost 52.3%, 54.7%, and 52.3% of their expected *CaCA* gene complements, respectively. A closer examination of *CaCA* gene duplication and deletion events, based on both duplication data and the phylogenetic tree, revealed that the 1:3:6 duplication ratio is not strictly maintained for any *AtCaCA* genes. In all cases, some *AtCaCA* orthologs were deleted in the *Brassica* species. Intriguingly, the *AtNCL1* gene lacks any detectable orthologs in *B. oleracea*, *B. rapa*, or *B. napus*, suggesting its complete deletion after the WGT event. This deletion could be due to either the lack of functional significance in the *Brassica* species or its replacement by genes with overlapping functions. The observed deletion of duplicated genes aligns with the concept of pseudogenization or non-functionalization, where one copy of a duplicated gene loses functionality over time and is eventually eliminated (Birchler and Yang, 2022). These findings align with previous studies indicating the existence of multiple *CaCA* gene copies in the angiosperm genome due to duplication events. The retention and loss of these genes are tightly linked to functional redundancy and differentiation (Zheng et al., 2021). Similar patterns of gene family member deletions in *Brassica* species have been documented for other families, including Tubby-like proteins (TLP), Diacylglycerol kinases (DGK), Metal Tolerance Proteins (MTP), and Lateral Organ Boundaries Domain (LBD) families (Tang et al., 2020; Wang et al., 2020; Xie et al., 2020, 2022) *B. rapa*, *B. oleracea*, and *B. napus* have lost 57%, 54%, and 57% of their TLP genes, respectively (Wang et al., 2020). Similarly, 52% of DGK genes have been deleted in *B. rapa*, 47% in *B. oleracea*, and 50% in *B. napus* (Tang et al., 2020). These results demonstrate the dynamic nature of plant genomes and their impact on the evolution of gene families through duplication and deletion.

### Identification of *cis*-regulatory elements of the CaCA superFamily

**Figure 7:**
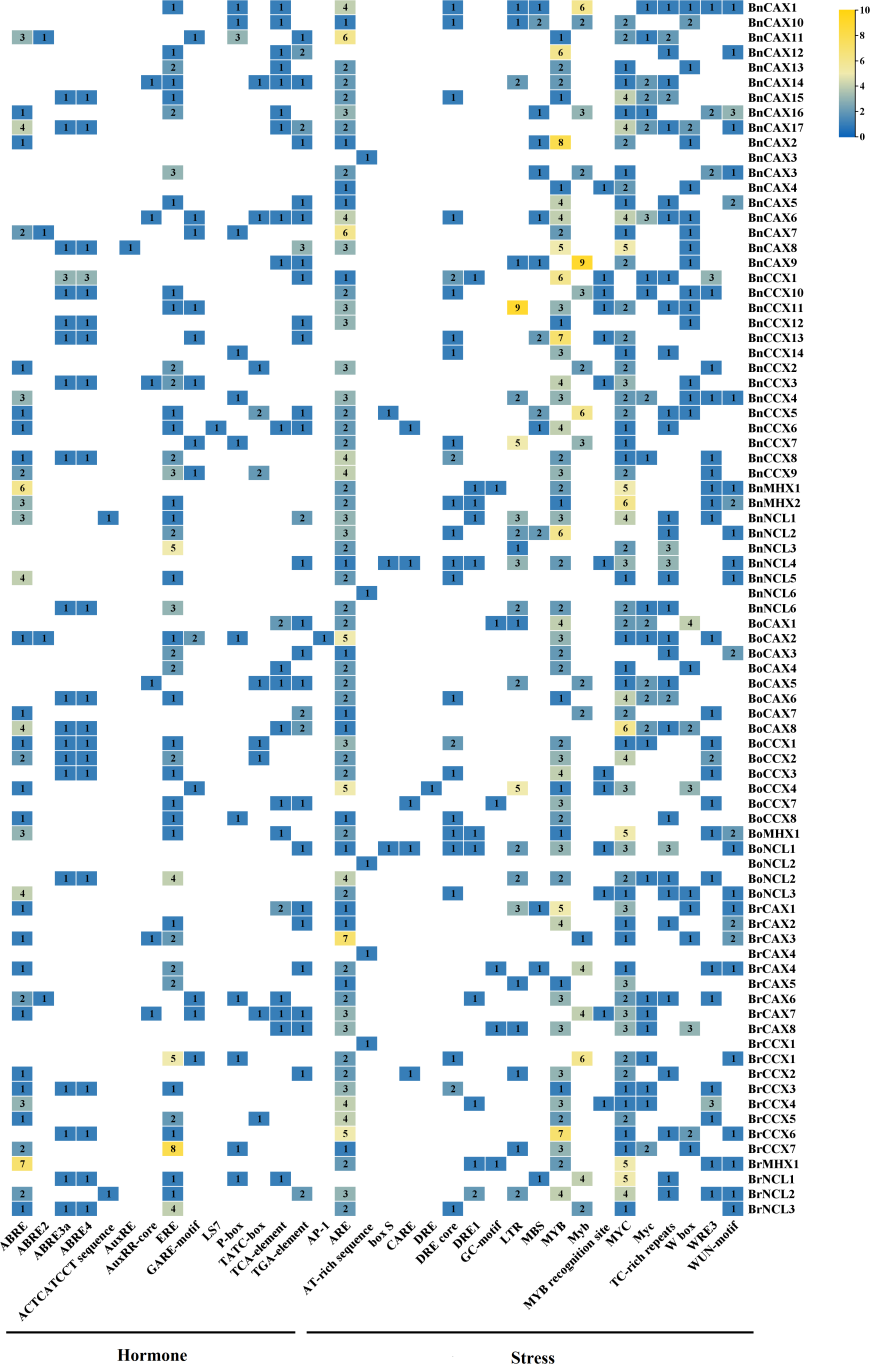
Promoter analysis (1.5 kilobases upstream of the start codon) of the CaCA superfamily in *B*. *napus*, *B*. *oleracea*, and *B*. *rapa*. Different colors and numbers within each box indicate the frequency of the corresponding cis-regulatory element in the promoter of that gene.

Promoter regions located upstream of genes are crucial for regulating transcription by RNA polymerase. These promoters harbor *cis*-regulatory elements that serve as binding sites for various transcription factors, orchestrating gene expression in response to developmental cues, environmental stresses, and physiological signals (Hernandez-Garcia and Finer, 2014). Recognizing the significance of *cis*-regulatory elements in defining gene function, we investigated the promoter regions of *CaCA* genes in *B. napus*, *B. oleracea*, and *B. rapa* to identify the associated *cis*-regulatory elements. The analysis revealed 14 distinct *cis*-regulatory elements within the promoters of these plants’ *CaCA* genes, responding to hormones such as ABA, ethylene, auxin, gibberellin, and salicylic acid (**Figure 7**, **Supplementary File 8**). These hormone-responsive elements occurred with a total frequency of 323. Additionally, the promoters contained 20 different *cis*-regulatory elements responsive to various stresses, with a total frequency of 877. These stress-responsive elements included those for cadmium, cold, drought, pathogens, wounds, elicitors, anaerobic stress, and anoxic conditions (**Figure 7**, **Supplementary File 8**). Among the hormone-responsive regulatory elements, ERF, ABRE, and TGA-element were most frequent, with frequencies of 85, 78, and 38, respectively. In contrast, stress-responsive elements such as ARE (172), MYB (162), and MYC (153) were most abundant (**Figure 7**, **Supplementary File 8**). Notably, all studied *CaCA* genes contained multiple stress- and hormone-responsive regulatory elements in their promoters, exhibiting varying frequencies (**Figure 7**, **Supplementary File 8**). For instance, in the promoter regions of the genes *BnNCL1*, *BoNCL2*, *BrCAX4*, and *BrCCX1*, only a single AT-rich sequence *cis-*element was identified. In contrast, the promoters of *BnCAC6* and *BrNCL2* contained 12 different types of *cis-*regulatory elements, totaling 24 occurrences (**Figure 7**, **Supplementary File 8**). Similar findings have been reported for CaCA gene promoters in other species, such as *M. domestica*, *Z. mays*, *Rosa roxburghii*, and *Populus trichocarpa* (Karami Lake et al., 2020; Mao et al., 2021; He et al., 2022; Zeng et al., 2024). For example, the promoters of *MdCaCA* genes were found to harbor a high frequency of regulatory elements responsive to cold, heat, drought, hypoxia, ABA, MeJA, auxin, ethylene, gibberellin, and salicylic acid (Mao et al., 2021). The presence of diverse *cis*-regulatory elements in the promoters of *CaCA* genes suggests that this gene family plays a pivotal role in plant adaptation to various environmental stresses and hormonal signals, as previously proposed (Sarcheshmeh et al., 2023). This highlights the intricate interplay between *cis*-regulatory elements and *CaCA* gene expression in mediating plant stress responses and hormonal regulation.

### Response of *BnCaCA* genes to abiotic stresses and ABA

**Figure 8:**
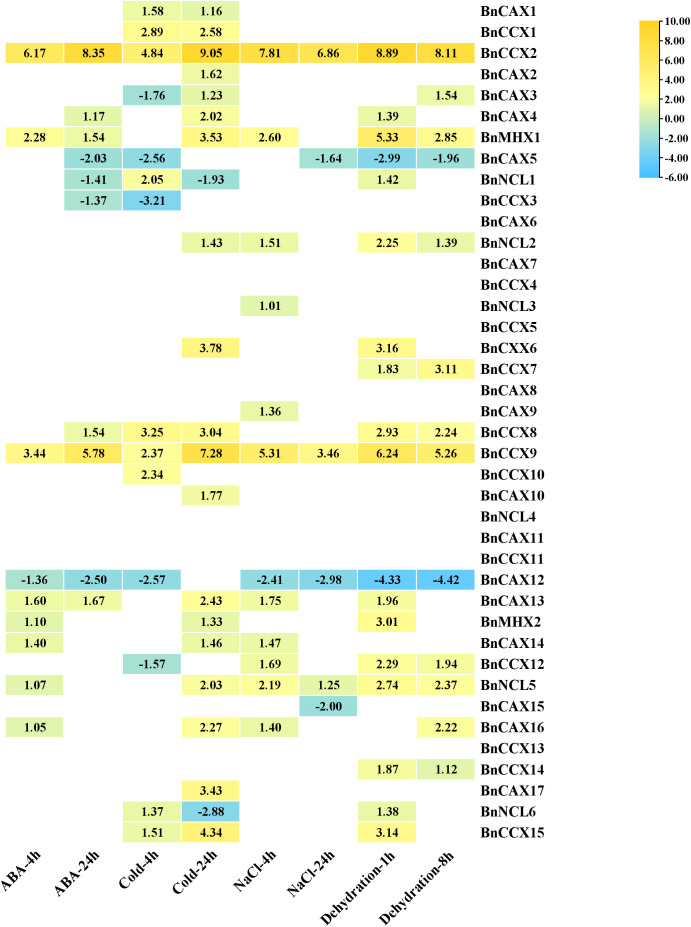
The expression pattern of *BnCACa* genes in response to ABA, Cold, NaCl, and dehyration stresses at various time points. Genes with |log2 (fold change)| > 1 and adjusted p-value < 0.01 were considered DEGs with significant expression changes. In the heat map, only the expression values of time points that showed a significant increase or decrease are displayed.

Changes in gene expression patterns under stress conditions and in response to hormones provide valuable insights into their functional roles in stress adaptation and hormone signaling pathways (Zhu et al., 2024). To elucidate this, we investigated the expression profiles of 40 *BnCaCA* genes in *B. napus* upon exposure to abiotic stresses (salinity, dehydration, and cold) and ABA treatment. The analysis revealed significant up-regulation or down-regulation of several *BnCaCA* genes in response to these stimuli. Notably, all genes except *BnCAX6*, *BnCAX7*, *BnCAX8*, *BnCAX11*, *BnCCX4*, *BnCC5*, *BnCCX11*, *BnCCX13*, and *BnNCL4* displayed significantly altered expression patterns under at least one treatment condition (**Figure 8**). These findings suggest that a subset of genes is involved in the response to each studied stress or hormone treatment. The least pronounced response was observed four hours after ABA application, with eight genes exhibiting significant induction and one gene showing repression (**Figure 8**). Conversely, the most significant response was observed 24 hours after cold stress application, with 19 genes up-regulated and two genes down-regulated (**Figure 8**).

By comparing gene expression patterns under various stress conditions, we identified two genes, *BnCCX2* and *BnCCX9*, as potential general stress response regulators within this family. Their expression was significantly up-regulated (4.8 to 9-fold for *BnCCX2* and 2.37 to 7.28-fold for *BnCCX9*) at all-time points across the studied treatments (**Figure 8**). The genes *BnMHX1* and *BnCAX13* were significantly induced in all conditions except for 4 hours after cold stress and 24 hours after salt stress, while *BnNCL5* was significantly induced in all conditions except for 24 hours after ABA treatment and 4 hours after cold stress (**Figure 8**). This suggests their crucial role in responding to abiotic stresses and ABA. Conversely, *BnCAX5* and *BnCAX12* exhibited down-regulation (-1.63 to -2.98-fold for *BnCAX5* and -1.35 to -4.41-fold for *BnCAX12*) in response to all treatments, suggesting their potential roles as negative regulators or in maintaining cellular homeostasis. Furthermore, analyzing expression patterns across different time points provides insights into the specific stress response phases. For instance, *BnCAX17*, *BnCAX10*, and *BnCCX6* displayed increased expression only at the 24-hour time point for cold stress, indicating their involvement in the late response phase (**Figure 8**).

The effect of stresses and hormones on the induction/repression of *CaCA* genes has been previously reported. For example, Analysis of the expression profile of *T. aestivum CaCA* genes in response to salt, heat, drought, and heat/drought stresses showed that the genes *TaCAX4-A*, *TaCAX4-B*, *TaCAX4-D*, and *TaCCX4-D* were significantly induced in response to all stresses, similar to *BnCCX2* and *BnCCX9*. This highlights the role of *CaCA* genes as key regulators of the stress response network. On the other hand, the gene *TaCCX3-D* is significantly repressed in response to abiotic stresses, and the genes *TaCAX1a-A*, *TaCAX1a-D*, *TaCAX1b-A*, and *TaCAX1b-D* are strongly repressed under salt stress conditions (Taneja et al., 2016). A study on *O. sativa* CAX genes showed that *OsCAX1a* and *OsCAX2* play roles as general signal transporters in growth, development, and stress response, while *OsCAX1b*, *OsCAX1c*, *OsCAX3*, and *OsCAX9* have evolved for specific stress response roles (Lian et al., 2024). Similar results have been observed in other plants such as *O. sativa*, *A. thaliana*, *M. domestica*, and *S.* sp*ontaneum* (Singh et al., 2015; Mao et al., 2021; Su et al., 2021).

Colinearity analysis identifies *BnCAX16* and *BnCAX3* as orthologs of Arabidopsis *AtCAX1* (*At2g38170*). Functional studies show that *AtCAX1* knockout mutants are hypersensitive to oxidative stress from methyl viologen and cadmium, accumulating more ROS (Baliardini et al., 2015; Ahmadi et al., 2018). Nitric oxide (NO) suppresses *AtCAX1* expression, and *AtCAX1* also participates in auxin signaling (Cho et al., 2012; Hussain et al., 2016). Transcriptomic data reveal that *BnCAX16* is strongly up-regulated by drought, salinity, cold, and ABA, while *BnCAX3* is induced by drought and cold. These results highlight a conserved role for these genes in plant responses to abiotic stress and hormones, likely through regulating ion homeostasis and protecting against oxidative damage. Their involvement in auxin signaling and NO regulation emphasizes their complex roles in environmental adaptation. Thus, *BnCAX16* and *BnCAX3* are promising candidates for breeding stress-resistant B. napus.

Additionally, *BnCCX5* and *BnCCX13* are orthologs of *AtCCX1* (*At5g17860*), and *BnCCX4* is an ortholog of *AtCCX2* (*At5g17850*). Studies show that *AtCCX1* promotes leaf senescence and modulates calcium signaling during aging (Li et al., 2016b). *AtCCX2* is induced by salt and osmotic stress; its knockout mutants have reduced tolerance to osmotic stress and impaired growth under salt, likely due to disrupted calcium flux (Corso et al., 2018) (Corso et al., 2018). However, in our studies, *BnCCX5*, *BnCCX13*, and *BnCCX4* showed no significant expression changes in response to abiotic stresses. This suggests that, while CCX members are important for stress and senescence in other plants, these genes may not directly mediate abiotic stress responses in *B. napus* under the tested conditions, or their roles may depend on specific stress types, timing, or tissues. Species-specific regulation likely accounts for these differences. The CaCA gene family’s defensive and regulatory roles are evident in other species. For example, *MdCCX1* and *MdCCX2* in apple are induced by salt stress and enhance salt tolerance by lowering sodium and increasing antioxidant activity (Yang et al., 2021a, b). These results indicate that *CaCA* genes play dynamic and multifaceted roles in plant stress adaptation, with both conserved and species-specific regulatory patterns.

## Conclusion

This study provides the first comprehensive evolutionary and functional analysis of the CaCA superfamily in *B. napus*, *B. rapa*, and *B. oleracea*, with Arabidopsis thaliana as a reference. We identified and classified 93 *CaCA* genes into four major clades (CAX, CCX, NCL, and MHX), revealed their gene structures and conserved motifs, and highlighted the impact of gene duplication and selection pressure on their diversification. Our expression profiling under abiotic stress conditions identified several *BnCaCA* genes (such as *BnCAX3*, *BnCAX16*, *BnCC2*, *BnCCX9*, *BnCAX5*, *BnCAX12*, *BnCAX13*, and *BnMHX1*) as potential candidates for stress tolerance breeding. However, a key limitation of this work is the absence of experimental validation for the predicted gene functions and stress responses. Future studies should focus on functional characterization of these candidate genes using genome editing tools such as CRISPR/Cas9 and the generation of transgenic lines to validate their roles in abiotic stress tolerance. Such efforts will not only confirm the bioinformatic predictions presented here but also accelerate the development of stress-resilient *Brassica* crops. Overall, our findings lay a solid foundation for molecular breeding and functional genomics studies aimed at improving crop resilience to environmental challenges.

## Data Availability

The original contributions presented in the study are included in the article/**Supplementary Material**. Further inquiries can be directed to the corresponding author.
