# Supplementary material for: Evolutionary dynamics of the calcium/cation antiporter superfamily in Brassicaceae: codon usage, selection pressure, and BnCaCAs role in abiotic stress response
Source: Front Plant Sci. 2025 Jul 8;16:1506461. doi: 10.3389/fpls.2025.1506461 (PMC12279817; doi:10.3389/fpls.2025.1506461)

**Supplementary File 4:** Conserved domain architecture of each sub-family within the CaCA gene family. Sequence logos represent the conserved motifs identified in the NCL, MHX, CAX, and CCX sub-families. Black boxes indicate the highly conserved α-repeat regions (α1 and α2 repeats), which are critical for ion transport function. Red boxes highlight the signature motifs characteristic of each sub-family. The EF-hand domain, involved in calcium binding, is also annotated where present. This figure illustrates both the conservation and divergence of key functional domains across the CaCA family sub-groups, providing insights into their structural and functional specialization.


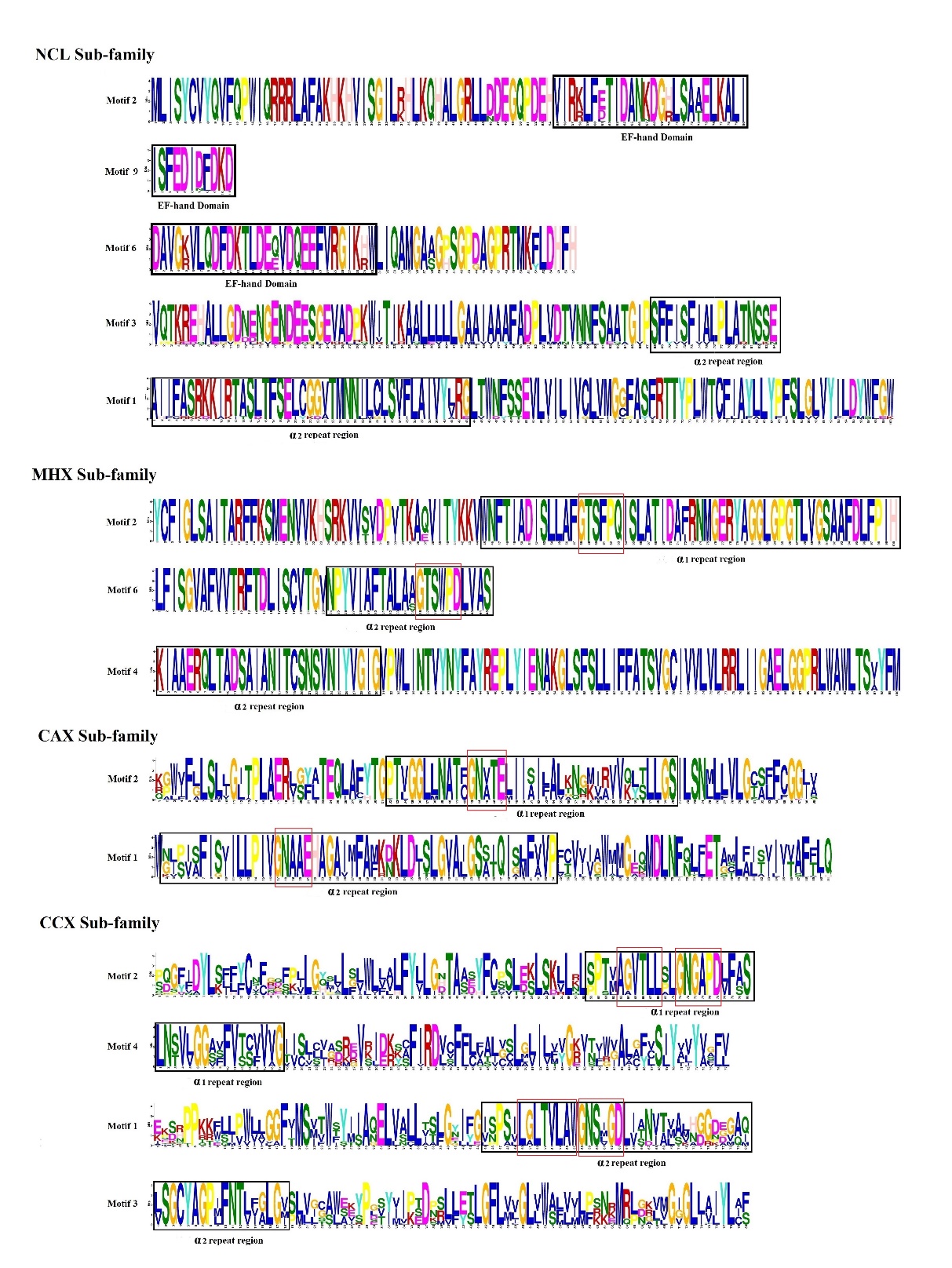

Supplement: Supplementary file 4 [file Table4.docx]
